# PLCγ-activated signalling is essential for TrkB mediated sensory neuron structural plasticity

**DOI:** 10.1186/1471-213X-10-103

**Published:** 2010-10-08

**Authors:** Carla Sciarretta, Bernd Fritzsch, Kirk Beisel, Sonia M Rocha-Sanchez, Annalisa Buniello, Jacqueline M Horn, Liliana Minichiello

**Affiliations:** 1 European Molecular Biology Laboratory, Mouse Biology Unit, Via Ramornie 32, 00015 Monterotondo, Rome, Italy; 2 Centre for Neuroregeneration, University of Edinburgh, EH16 4SB Edinburgh, UK; 3 Creighton University, Department of Biomedical Sciences, Omaha, NE, 68718, USA; 4 University of Iowa, Department of Biology, Iowa City, IA, 52242 -1324, USA; 5 Actelion Pharmaceuticals, Ltd., Gewerbestrasse 16, CH-4123 Allschwil, Switzerland

## Abstract

**Background:**

The vestibular system provides the primary input of our sense of balance and spatial orientation. Dysfunction of the vestibular system can severely affect a person's quality of life. Therefore, understanding the molecular basis of vestibular neuron survival, maintenance, and innervation of the target sensory epithelia is fundamental.

**Results:**

Here we report that a point mutation at the phospholipase Cγ (PLCγ) docking site in the mouse neurotrophin tyrosine kinase receptor TrkB (Ntrk2) specifically impairs fiber guidance inside the vestibular sensory epithelia, but has limited effects on the survival of vestibular sensory neurons and growth of afferent processes toward the sensory epithelia. We also show that expression of the TRPC3 cation calcium channel, whose activity is known to be required for nerve-growth cone guidance induced by brain-derived neurotrophic factor (BDNF), is altered in these animals. In addition, we find that absence of the PLCγ mediated TrkB signalling interferes with the transformation of bouton type afferent terminals of vestibular dendrites into calyces (the largest synaptic contact of dendrites known in the mammalian nervous system) on type I vestibular hair cells; the latter are normally distributed in these mutants as revealed by an unaltered expression pattern of the potassium channel KCNQ4 in these cells.

**Conclusions:**

These results demonstrate a crucial involvement of the TrkB/PLCγ-mediated intracellular signalling in structural aspects of sensory neuron plasticity.

## Background

The vestibular system consists of 5 end organs, which include 3 semicircular canals and their associated cristae involved in angular acceleration, and two otolith organs (the utricle and the saccule) involved in linear acceleration, including responses to gravity. Each of these end organs contains a sensory epithelium, which in mammals consists of two types of hair cells, namely type I and type II (50% each) that have distinct distribution patterns, polarity and physiology [1]. *Bdnf *is expressed in both types of differentiated hair cells in each of the sensory organs during embryonic and neonatal inner ear development [2].

Vestibular sensory neurons reside in the vestibular ganglia and are bipolar, sending a peripheral process (afferent) to the hair cells in their respective sensory epithelia, and a central process to the vestibular nuclei of the medulla [3]. Two different types of afferent fibers innervate the hair cells. Thick fibers innervate type I hair cells and form large calyceal endings, whereas thin fibers innervate type II hair cells and exclusively form bouton terminals. It has been clearly shown by studies of genetic mutations in mice that in the inner ear (which comprises the vestibular and cochlear systems) vestibular sensory neuron survival and innervations mainly depend on the neurotrophin receptor tyrosine kinase TrkB and its ligand BDNF [4].

BDNF/TrkB activates three main intracellular signalling cascades. The Ras/mitogen activated protein kinase (MAPK) pathway and the phosphoinositide 3-kinase (PI3K) pathway are activated primarily through SHC/FRS-2 binding to phosphorylated tyrosine (Y)515, whereas phosphorylated Y816 activates a calcium/calmodulin-dependent protein kinase II (CaMKII) pathway through PLCγ. We have previously described the creation of highly defined mouse models that carry tyrosine to phenylalanine (F) point mutations at either Y515 (TrkB^SHC ^mice) or Y816 (TrkB^PLC ^mice). Using these genetic models, we have been able to show that in the central nervous system the PLCγ docking site is necessary for TrkB-mediated hippocampal synaptic plasticity [5]. Moreover, a TrkB Y816F but not Y515F point mutation impairs both the acquisition of an associative learning task and long-term potentiation (LTP) between hippocampal neurons *in vivo *[6]. Recently, we have also shown that the Y816F point mutation in TrkB impairs acquisition of fear learning, amygdalar synaptic plasticity, and CaMKII signalling at synapses. In contrast, a Y515F point mutation affects consolidation but not acquisition of fear learning to tone, and also alters AKT activation [7]. In the peripheral nervous system, we have previously reported that a point mutation in the SHC binding site of TrkB only mildly affects vestibular sensory neuron survival, indicating that TrkB receptors promote long-term survival of sensory neurons mainly in a SHC site-independent manner [8]. In contrast, fiber growth was reduced, and target innervation by these sensory neurons was ultimately lost in TrkB^SHC ^mice [9]. These results suggested that TrkB receptor signals that maintain target innervation require the SHC site. Here we have asked whether the PLCγ docking site of TrkB would be responsible for the fiber guidance role of TrkB reported in the inner ear [10], possibly through activation of TRPC channels as recently demonstrated *in vitro *[11,12]. We find that the major function of the PLCγ site downstream of TrkB is fiber guidance, but surprisingly only inside the vestibular sensory epithelia, with limited effects on the survival of vestibular sensory neurons and growth of afferent processes toward the sensory epithelia. We also demonstrate that double point mutations in both the SHC and the PLCγ docking sites (referred to as TrkB^D ^mice) [13] results in a loss of vestibular neurons that is essentially indistinguishable from that of *Trkb *null mice (*Trkb^-/-^*), suggesting that the survival function of BDNF/TrkB is mediated predominantly through the cooperative action of these two docking sites, whereas the PLCγ site-activated signalling pathway(s) of TrkB is essential for the proper intraepithelial navigation of fibers. In addition, we show that the transformation of afferent dendritic boutons into calyces requires BDNF/TrkB signalling via the PLCγ docking site, revealing the requirement of this site for structural plasticity of sensory neurons.

## Results

### Vestibular neurons survive in the absence of a functional TrkB PLCγ site, but not in absence of both the PLCγ and the SHC sites

**Figure 1:**
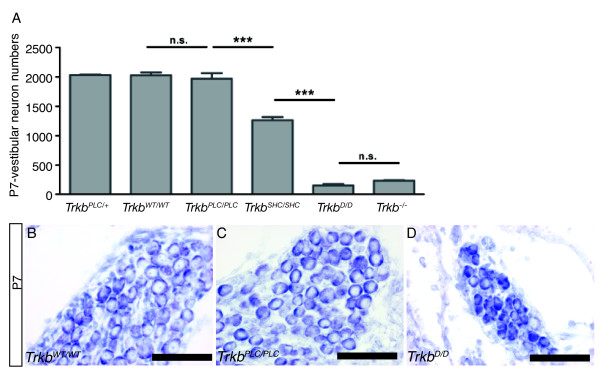
**Vestibular neurons survive in absence of a functional TrkB/PLC**γ **site, but nearly all die by disrupting both the TrkB/PLC**γ **and SHC sites**. (A) Total vestibular neuron numbers at P7 in *Trkb *point mutants (*Trkb^PLC/PLC^*; *Trkb^D/D^*) compared to controls (*Trkb^WT/WT^*; *Trkb^PLC/+^*). Shown also is the loss of vestibular neurons in *Trkb^SHC/SHC ^*point mutants and in *Trkb *null mice (*Trkb^-/-^*) for comparison. (B-D) Representative cresyl violet-stained horizontal sections of the central portion of the vestibular ganglion of control and point mutant (*Trkb^PLC/PLC^*; *Trkb^D/D^*) mice at P7 as indicated. ***, p < 0.0001 (ANOVA). Scale bar, 50 μm.

**Figure 2:**
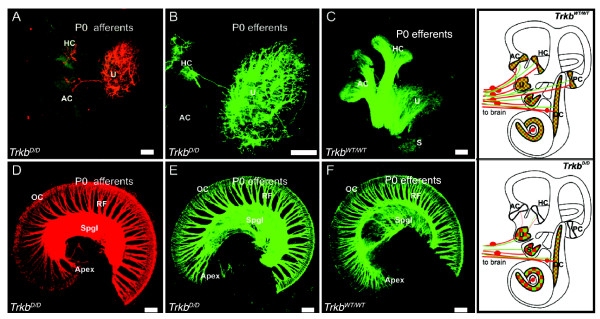
**Innervations of vestibular and cochlear sensory epithelia are highly compromised in mice lacking both the PLC**γ **and the SHC docking sites of TrkB**. The pattern of afferent (A, D) and efferent (B-C, E-F) innervations of newborn control (C, F) and *Trkb^D/D ^*mutant mice (A-B, D-E) is revealed by filling afferent or efferent from specific areas of the brainstem. Note that many afferent (A) and efferent (B) fibers reach the utricle (u) but only few fibers reach the canal cristae (A, B), whereas canal cristae are densely innervated in control mice (C). *Trkb^D/D ^*mutant mice show wider spacing between radial fiber bundles (RF), both afferent (D) and efferent (E), compared to control (F). Schematic diagrams show the changes in innervation density of afferents (red) and efferent fibers (green) to the various sensory epithelia. Note that in *Trkb^D/D ^*mutant mice compared to controls (*Trkb^WT/WT^*) the fiber density to canal cristae is reduced (AC, HC) or lost (PC) and that the density of innervation of utricle, saccule and cochlear apex is also reduced (larger green/red boxes), whereas the basal turn organ of corti innervation density is similar to controls. Innervation density in the schematic diagrams is indicated by green/red squares. AC, anterior cristae; HC, horizontal cristae; U, utricle; S, saccule; OC, organ of Corti; RF, radial fibers; Spgl, spiral ganglion. Scale bars, 100 μm.

Previously we have shown that a point mutation at the SHC site of TrkB only partially affects the survival of inner ear vestibular neurons (25% loss) compared to control mice at postnatal day 7 (P7) [8]. Neurons remaining at P7 survived into adulthood, indicating that activation of pathways independent of the SHC site support the survival of vestibular neurons given that in the *Trkb^-/- ^*mutants the majority of these neurons are already lost at birth [8,9]. Vestibular ganglia of *Trkb^PLC/PLC ^*mutants, which are homozygous for the Y816F point mutation, and control mice (*Trkb^WT/WT^*, *Trkb^PLC/+^*) were analysed to determine if the PLCγ site of TrkB supports vestibular neuron survival. Neuronal counts revealed a comparable number of sensory neurons in the vestibular ganglion of *Trkb^PLC/PLC ^*mutants at P7 compared to the control genotypes (*Trkb^WT/WT^*, and *Trkb^PLC/+^*) (Fig. 1A). We then determined whether the two tyrosines comprising the SHC and the PLCγ docking sites together mediate the functions of *Trkb *in the vestibular ganglion. Homozygous double point mutant mice (*Trkb^D/D^*)[13] showed a loss of vestibular neurons equivalent to that of *Trkb^-/- ^*mice (Fig. 1A-D) [14], and had a complete lack of afferent innervations to the posterior canal cristae with only an occasional fiber projecting to the anterior and horizontal cristae (Fig. 2A). The innervations to the utricle and saccule were reduced and closely resembled those of *Bdnf^-/- ^*mice [15] and of *Trkb^-/- ^*mice [16] previously reported. Similar effects were also obtained for the efferent innervations to the ear (Fig. 2B,C).

Previously, it was shown that deletion of *Trkb *or *Bdnf *reduces radial fiber density in the apex of the cochlea [16], a region that normally expresses high levels of BDNF during development [17]. Mice carrying a point mutation at the PLCγ site of TrkB did not show any deficits in the cochlea (data not shown), and only mild defects were found in the cochlea of the *Trkb^SHC/SHC ^*point mutants [9]. In contrast, mice lacking both the PLCγ and the SHC docking sites of TrkB had an obvious reduction in radial fiber density in the cochlea (Fig. 2D, E) compared to control mice (Fig. 2F). These data strongly support the idea that the intracellular signalling of TrkB predominantly depends on these two docking sites, as the vestibular and cochlear phenotypes resulting from their mutation produces a virtually identical phenotype to that seen in *Trkb *null mutant animals.

### TrkB/PLCγ signalling is necessary for fiber guidance in the sensory epithelium

**Figure 3:**
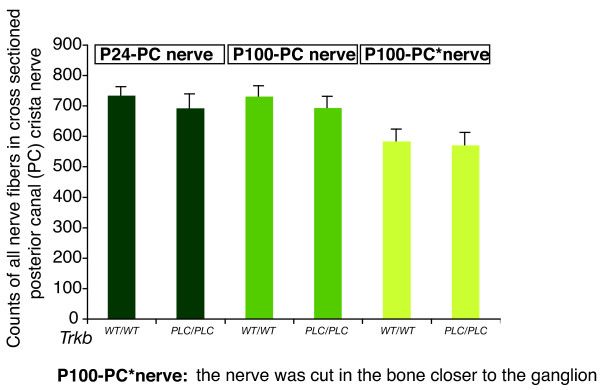
**Number of myelinated nerve fibers in the posterior canal (PC) nerve**. Counts of all myelinated nerve fibers in the posterior canal nerve at P24 and P100 did not show a difference in the number of nerve fibers between *Trkb^PLC/PLC ^*mice and control animals. The PC*nerve was cut in the bone closer to the ganglion.

**Figure 4:**
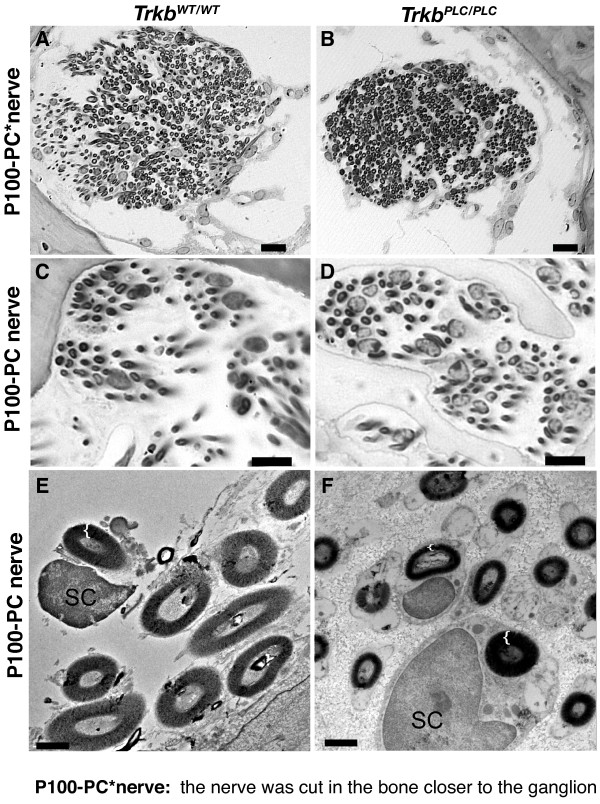
**The PLC**γ site **of TrkB is involved in the maturation of nerve fibers**. (A-D) Cross sections of P100 nerves to the posterior cristae of *Trkb^PLC/PLC ^*and aged matched control mice are shown. (E-F) TEM images from P100 nerves to the posterior cristae; note that *Trkb^PLC/PLC ^*mice show similar number of nerve fibers compared to the control animals, but nerve fibers tend to be much smaller in diameter with less myelin. Left brace "{" indicates the thickness of myelin in E-F panels and shows that reduction varies between fibers of comparable diameter of the neuronal process. Scale bars, 100 μm in A-D, 1 μm in E, F.

Previously, counts of nerve fibers to a single vestibular sensory epithelium (the posterior canal cristae, PC) showed that *Trkb^SHC/SHC ^*point mutant mice had reduced fiber growth with the remaining fibers exhibiting a reduction in nerve diameter and loss of myelin [9]. We carried out a similar analysis in *Trkb^PLC/PLC ^*mutants by counting all nerve fibers to the PC sensory epithelium and found no differences in the number of nerve fibers apparent in juvenile or adult mutant mice, compared to controls (Fig. 3, 4. However, in the *Trkb^PLC/PLC ^*mice there was a slight reduction in the degree of myelination (Fig. 4C), which was particularly apparent by transmission electron microscopy analysis (TEM) (Fig. 4E-F). These data suggest that the PLCγ signalling downstream of TrkB is not required for vestibular neuron fiber growth or maintenance, but rather functions in certain aspects of fiber maturation pertaining to their degree of myelination.

We next investigated nerve fiber innervation toward and within the vestibular sensory epithelia. The afferent fibers were targeted to a given sensory epithelia in both the *Trkb^PLC/PLC ^*point mutant and the control animals at P0 and P8, showing no fibers outside the sensory epithelia (data not shown). Nerve fibers targeting the vestibular sensory epithelia were also observed in the *Trkb^SHC/SHC ^*point mutants [9], and in the *Trkb^D/D ^*point mutants (Fig. 2). Thus, nerve fiber targeting to the sensory epithelia mediated by BDNF [18] must use signalling mechanisms independent of the TrkB/SHC or PLCγ sites and/or factors other than BDNF/TrkB signaling such as NT3/TrkC.

**Figure 5:**
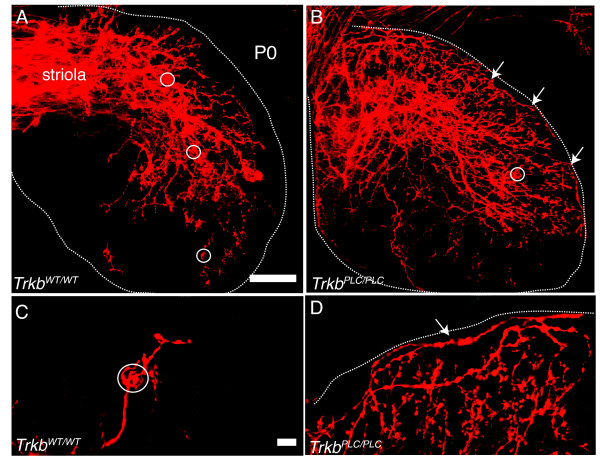
**Afferent fibers are disorientated inside the vestibular sensory epithelia in absence of the TrkB/PLC**γ **docking site**. (A-D) Shown are afferent fibers to the utricle labelled from the cerebellum; (A, C), P0 *Trkb^WT/WT ^*control, and (B, D) P0 *Trkb^PLC/PLC ^*point mutants. Note the highly focused projection in the control mice that already displays partial calyx formation (outlined by circles, A, C) with no fibers extending along the epithelial perimeters (dotted outline). In contrast, *Trkb^PLC/PLC ^*mice show fibers extending for long distances along the epithelial perimeters (dotted outline) as well as within the sensory epithelium (arrows in panels B, D). There is only an occasional indication of partial calyx formation in *Trkb^PLC/PLC ^*mutants at this stage (B, white circle). Scale bars, 100 μm in (A, B) and 10 μm in (C, D).

Closer examination of the nerve fiber trajectories within the sensory epithelia showed aberrations in the *Trkb^PLC/PLC ^*point mutant mice that were not found in the *Trkb^WT/WT ^*control mice (Fig. 5) nor in the *Trkb^+/+ ^*mice (data not shown). As early as P0, the *Trkb^WT/WT^*, like the *Trkb^+/+^*, control mice showed obvious targeting of nerve fibers toward hair cells and already partial formation of calyces (Fig. 5C). Conversely, in *Trkb^PLC/PLC ^*point mutants, nerve fibers extended for long distances along the perimeter of sensory epithelia (Fig. 5B, D). It is noteworthy that these fibers did not leave the sensory epithelia despite their obvious disorientation. In addition, at this stage these nerve fibers rarely engaged in the formation of calyces as compared to the *Trkb^WT/WT ^*controls (Fig. 5C).

**Figure 6:**
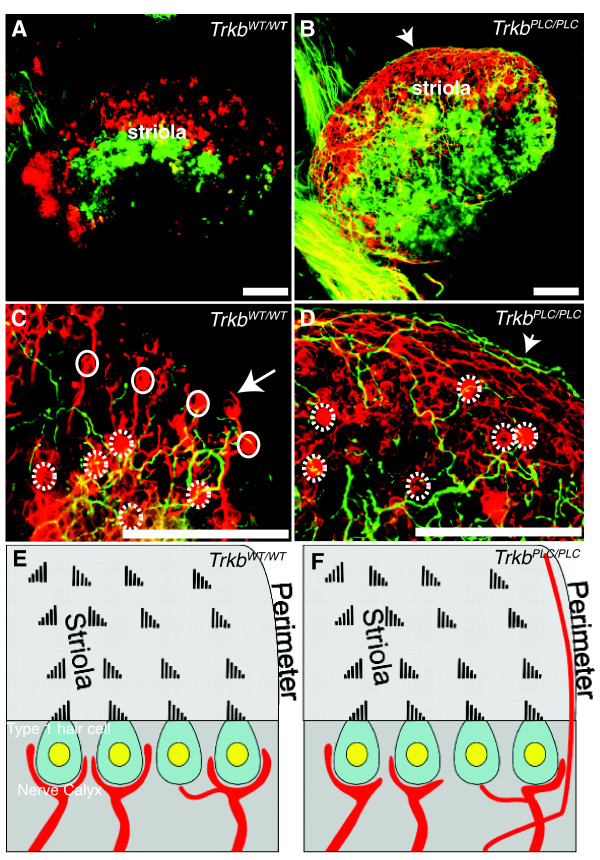
**Abnormal calyx formation and hair cell innervations in absence of a functional TrkB/PLC**γ **site**. (A-F) P8 utricular afferent projections labelled with lipophilic dye injected either into the cerebellum (red) or the brainstem (green) were compared. Note that the overall sorting of afferent fibers within the utricle is comparable between the *Trkb^PLC/PLC ^*mutant mice and controls. However, many more fibers overlap in the *Trkb^PLC/PLC ^*mutants compared to control (yellow in A, B). (C) Closer examination shows numerous hair cells surrounded by partial or complete calyces (indicated by dashed white circles in the striola region) in *Trkb^WT/WT ^*controls, white circles indicate those near the edge of the striola region that are found only in *Trkb^WT/WT ^*control mice. White arrow points to a peripheral calyx with nerve fiber coming off (so called mixed calyx/bouton fiber in the non-striola region). (D) Note absence of calyces outside the striola region in *Trkb^PLC/PLC ^*mutants, and only occasional calyces inside the striola region of the *Trkb^PLC/PLC ^*mutant mice (indicated by dashed white circles). Moreover, the long distances fibers are running inside the sensory epithelium as well as along the perimeter in the *Trkb^PLC/PLC ^*mutant mice (arrowhead) (B, D), whereas much shorter and branched trajectories are typical in control mice (C). (E-F) Schematic drawings showing hair cell innervation within the striola region of the utricular sensory epithelium (E-F); the striola region is characterized by polarity reversal of hair cells and the presence of calyces around type 1 hair cells. Note that calyces in *Trkb^PLC/PLC ^*mutants tend to be smaller, less frequent in the striola region and incomplete or absent near the perimeter (F); whereas *Trkb^WT/WT ^*controls form calyces both inside and outside of the striola region (E). Moreover, unique to *Trkb^PLC/PLC ^*mutants are afferent fibers that run along the perimeter of the sensory epithelia for long distance. Scale bars, 100 μm.

We further investigated calyx development at P8, a stage when most calyces are present [19,20]. Labeling of afferent fibers from the cerebellum and the brainstem showed distinct groups of labelled fibers in control mice, apparently situated on either side of the striola region (Fig. 6A). Interestingly, *Trkb^PLC/PLC ^*point mutant mice showed a comparable distribution of fibers labelled from the cerebellum and the brainstem as control mice (Fig. 6B), however, two major differences were immediately apparent even at low magnification. Firstly, many fibers in the *Trkb^PLC/PLC ^*mutants crossed into the vicinity of the other fiber compartments and circled along the sensory epithelia perimeter, occasionally for several 100 μm (Fig. 6B, D). Secondly, despite the apparently normal fiber density, the calyces detected in the *Trkb^PLC/PLC ^*point mutants were almost exclusively inside the striola region (Fig. 6D), whereas controls at P8 showed numerous calyces and good segregation of nerve fibers into discrete territories up to the perimeter of the utricle (Fig. 6C). Only a few fibers were near the margin of the sensory epithelium, and the fibers showed numerous branches consistent with their innervating type II hair cells [21]. In contrast, *Trkb^PLC/PLC ^*point mutants displayed little fiber segregation, with both those at the margin and within the epithelium broadly overlapping (Fig. 6B, D). Similar effects were found in the other four vestibular epithelia (data not shown). Comparable results were also observed in older mutants (P24) and control mice (data not shown). This phenotype results in impaired balance in *Trkb^PLC/PLC ^*mutants compared to control mice. The *Trkb^PLC/PLC ^*mice showed a higher basal locomotor activity and displayed circling behavior, in either direction, compared to wild-type control mice (*Trkb^WT/WT)^*) (Additional files 1 &2).

Overall, these data suggest that a point mutation at the PLCγ site of TrkB causes disorientation of afferent fibers inside the vestibular sensory epithelia. In addition, nerve fibers either do not find type I hair cells or cannot engage in calyx formation, as a reduction in both the distribution and the degree of differentiation of calyces is apparent.

### Ultra structural analysis confirms the inability of calyx formation in *Trkb^PLC/PLC ^*point mutants

**Figure 7:**
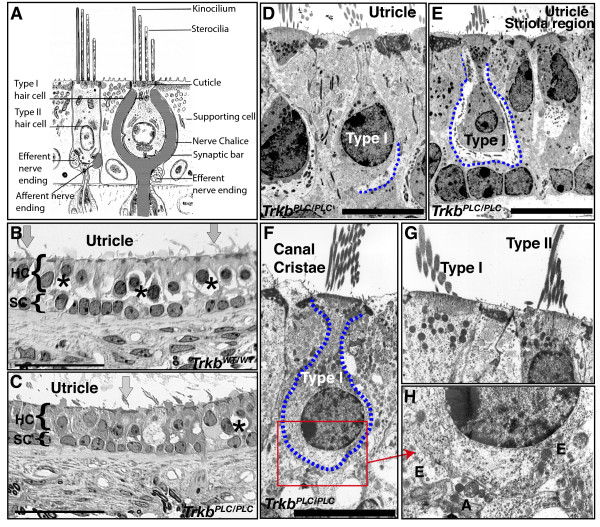
**Histological evidence for the inability of calyx formation in *Trkb^PLC/PLC ^*point mutants**. (A) Adapted scheme showing major differences between type I and type II hair cells. (B-C) Light microscopy pictures showing the appearance of utricular sensory epithelia at postnatal day 100 with large calyceal spaces around type I hair cells in the *Trkb^WT/WT ^*control (asterisks) (B), compared to little calyx formation in the *Trkb^PLC/PLC ^*mutant mice (asterik) (C); arrows in both panel B and C indicate the stereocilia suggesting that hair cells are normally developed. Braces "{" indicate the supporting cells (SP), or the hair cells (HC). (D-E) Electron microscopy (EM) micrographs showing a typical type I hair cell in the utricle of *Trkb^PLC/PLC ^*mutant mice mostly without a fully formed calyx *in vivo*. However, in the striola region there is an occasional type I hair cell with a fully formed calyx (E) in the utricle of *Trkb^PLC/PLC ^*mutant mice. (F) A canal cristae type I hair cell of the *Trkb^PLC/PLC ^*mutants innervated by several afferent only forming partial calices and efferent boutons (H). The existence of two types of hair cells is verified by the examination of hair cell apices, which show the presence of two different sizes of stereocilia and mitochondria but no calyx (G) as previously described [19,47]. Scale bars, 100 μm (panels B-C), 10 μm (panels D-F), (G-H are twice the magnification of F).

We next investigated the formation of calyces in histological sections of P24 and P100 control and *Trkb^PLC/PLC ^*point mutants. Histological sections clearly revealed formation of calyces in all vestibular epithelia of control mice already at P24 (data not shown, and Fig. 7B, P100, utricle), as previously described [20,22]. In contrast, *Trkb^PLC/PLC ^*mutants showed little evidence of calyx formation in any vestibular sensory epithelium (Fig. 7C, P100, and data not shown). Likewise, electron microscopy (EM) analysis showed limited and mostly partial calyx formation throughout the vestibular epithelia of *Trkb^PLC/PLC ^*mutants, in particular in the utricle (Fig. 7C, D), with the exception of an occasional calyx-like structure around the striola region of the utricle (Fig. 7E). In the canal cristae calyx formation was almost absent and only formation of boutons was apparent (Fig. 7F and insets Fig. 7H), indicating that the conditions for calyx formation inside and outside of the striola region and between sensory epithelia differ.

### *Trpc3 *is overexpressed in vestibular organs of *Trkb^PLC/PLC ^*mutants and may lead to the fiber misdirection

**Figure 8:**
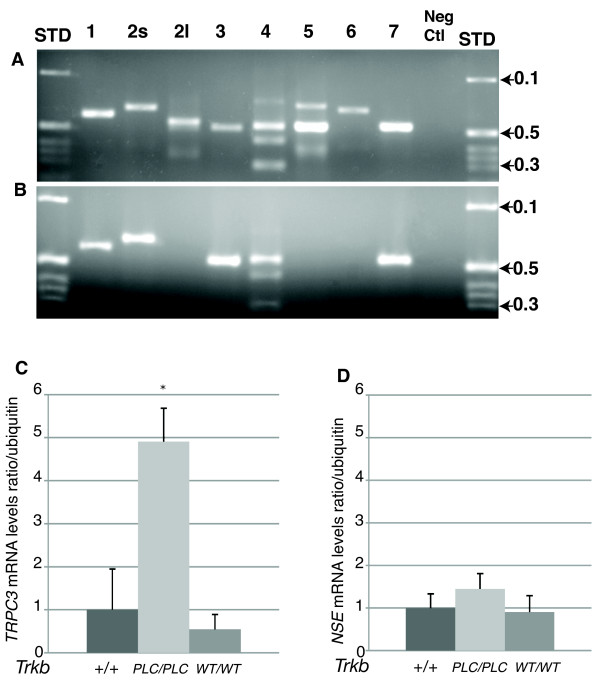
**TRPC3 is upregulated in *Trkb^PLC/PLC ^*mutants**. (A-B) *Trpc *isoform expression analysis in mouse adult vestibular tissue. Expression analysis of *Trpc1-7 *isoforms was performed by RT-PCR using as control total RNA preparations from a pool (A) consisting of E15 brain and retina and dissected vestibular ganglion tissue (B). All PCR products were verified by direct sequence analyses. The negative control was a PCR reaction with the total RNA template without the addition of reverse transcriptase. A 1 kb ladder was used as a standard size marker (STD) and the corresponding kb lengths were indicated. (C) RT-PCR for *Trpc3 *showed increased expression of this channel in the vestibular organ of *Trkb^PLC/PLC ^*mutants compared to both controls (wt and *Trkb^WT/WT^*, p = 0.02). No difference was detected between the two controls (p = 0.5). (D) Neuron specific enolase (NSE) was used as an internal control specific to neurons. No significant difference was observed between the *Trkb^PLC/PLC ^*mutants and the two controls.

BDNF signalling has been shown to induce chemo-attractive turning of axonal growth cones *in vitro *[23]. Moreover, it has also been shown that BDNF-induced guidance of nerve growth cones in rat cerebellar granule cell cultures depends on the activation of signalling downstream of the PLCγ site of TrkB and Ca^2+ ^elevation [11]. TRPC channels, in particular TRPC3/6, mediated the BDNF-induced elevation of Ca^2+ ^in these cells, and were required for the nerve-growth cone turning induced by BDNF [11]. Because individual fibers inside the sensory epithelia showed impaired guidance in *Trkb^PLC/PLC ^*mutants compared to controls (Fig. 6E-F), we decided to investigate the involvement of TRPC channels in fiber misdirection in the vestibular sensory epithelia of *Trkb^PLC/PLC ^*mutants. We first examined the expression of *Trpc1-7 *by RT-PCR analyses using total RNA preparations from dissected vestibular ganglion tissue of young adult CF-1 male mice (4-5 weeks old). Positive control samples consisted of cDNA templates derived from a pool of total RNAs prepared from mouse embryonic day 15 (E15) brain and retina. As shown in Fig. 8A-B, *Trpc1*, *2s*, *3*, *4*, and *7 *are expressed in both vestibular tissue and E15 brain/retina. Since TRPC3/6, but not TRPC1, has been shown to be essential in the guidance of nerve growth cones by BDNF [11], we examined the expression of T*rpc*3 in the vestibular organ of *Trkb^PLC/PLC ^*mutants and control (*Trkb^+/+ ^*and the *Trkb^WT/WT^*) mice. As shown in Fig. 8C, T*rpc*3 was overexpressed in the *Trkb^PLC/PLC ^*mutants compared to controls (one-way ANOVA, p = 0.02). Moreover, mRNA levels of the neuronal marker neuron-specific enolase (NSE) were not significantly different between the *Trkb^PLC/PLC ^*mutants and the control genotypes (Fig. 8D). We also anlysed *Trpc4 *channel expression by RT-PCR and found no differences between all genotypes analysed (data not shown).

### KCNQ4 shows normal distribution in *Trkb^PLC/PLC ^*mutants

**Figure 9:**
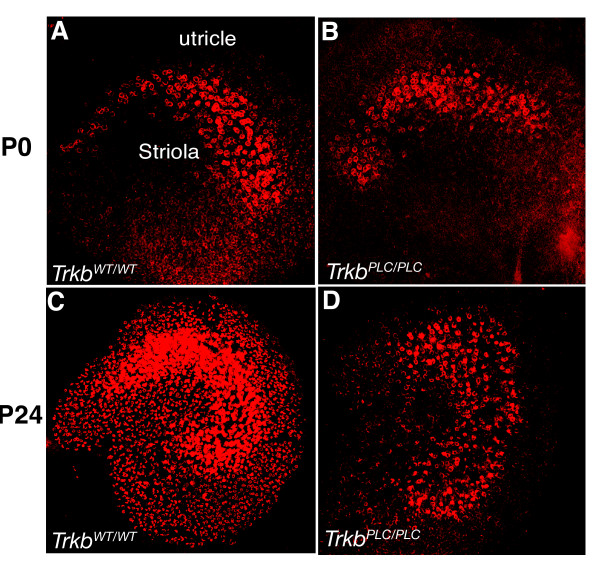
**Calyx formation does not regulate expression patterns of KCNQ4**. (A-D) Show the expression analysis of KCNQ4 in control (*Trkb^WT/WT^*) and *Trkb^PLC/PLC ^*mutant mice at two different stages, P0 and P24. Upregulation of KCNQ4 protein shows only minor quantitative differences between control and *Trkb^PLC/PLC ^*mutant. Scale bars, 100 μm.

KCNQ4 is an M-type K^+ ^channel expressed in sensory hair cells of the inner ear and in the central auditory pathway. Previous work had suggested that *Kcnq4 *expression is restricted to the vestibular afferent calyx-like nerve endings ensheathing the type I hair cells [24]. However, an analysis of conditional *Bdnf *null mutant mice has revealed that type I hair cells express *Kcnq4 *in the absence of afferent and/or efferent innervations in the vestibular canal cristae [25]. Immunohistochemical analyses revealed no differences in the expression pattern of *Kcnq4 *in the canal cristae and the utricle of *Trkb^PLC/PLC ^*and control mice at both P0 and P24 (Fig. 9A-D). Therefore, the distribution of type I hair cells is normal at a time (P0) when calyx formation is already affected in the absence of the PLCγ site of TrkB. By P24, the intensity of the immunohistochemical response of KCNQ4 in the tissue from *Trkb^PLC/PLC ^*mutants compared to control tissue was reduced (Fig. 9A-D), suggesting that calyx formation does not regulate the expression pattern of KCNQ4, but its expression level in type I hair cells is influenced by proper innervation.

## Discussion and Conclusions

The neurotrophin receptor tyrosine kinase TrkB is primarily known for its function during peripheral and central nervous system development [26]. In the CNS it is only recently emerging as a potent regulator of synaptic plasticity of the hippocampus as well as of other brain regions, and the signalling pathways and molecular mechanisms involved in these functions are beginning to be revealed [7,27]. As for the peripheral nervous system, in order to dissect the signalling pathways involved in the predominant functions of TrkB receptors, such as neuronal survival as well as target innervation, we have chosen the vestibular organ as a model. We have previously reported that a point mutation at the SHC site of TrkB has limited effects on vestibular sensory neuron survival, indicating that TrkB receptors promote long-term survival of sensory neurons mainly in a SHC site-independent manner [8]. In contrast, target innervations of these sensory neurons were eventually lost in TrkB^SHC ^mice; at P0 both afferent and efferent fibers were evident to all sensory epithelia, however by P8 they were greatly reduced [9], revealing that signalling through the SHC site of TrkB is required to maintain target innervations. However, no molecular data has been known until now concerning the growth of vestibular fibers toward specific vestibular hair cell types and, in the saccule and utricle, toward hair cells of different polarity. It was recently shown that fibers targeting hair cells of different polarity have distinct central projections [28], but the mechanisms underlying these features are unclear. We now show that in the absence of the PLCγ docking site of TrkB the apparent segregation of fibers from distinct central projection areas is partially disrupted. In particular, we show that while the growth of vestibular afferents to inner ear sensory epithelia are unaffected in TrkB^PLC ^mutants, these fibers run along the peripheral margin of the sensory epithelia without taking sharp turns to innervate their specific hair cells as in control mice (Fig. 5,6). Thus, signalling elicited through the PLC-γ site of TrkB is involved in targeting processes inside the sensory epithelia. This is consistent with previous reports showing that targeted growth to sensory epithelia does not require neurotrophins [17,29], does not require differentiated hair cells [18,30], and can occur in the absence of sensory epithelia formation [31]. Moreover, it is the cooperation of both the SHC and the PLCγ docking sites of TrkB [13] that mediate the survival of the vestibular neurons, as the phenotype of the TrkB^D ^mice with respect to the loss of these cells is essentially indistinguishable from that observed in *Trkb *null mice.

The TRPC3/6 calcium channels have been shown to mediate the BDNF-induced elevation of Ca^2+ ^in rat cerebellar granule cell cultures and to be required for the nerve-growth cone turning induced by BDNF in these cells [11]. Here we show that TRPC3 is overexpressed in vestibular neurons of TrkB^PLC ^point mutants. Overexpression of the *Trpc3 *channel has been reported to interfere with the cellular calcium homeostasis both *in vitro *and *in vivo *[32,33], this could explain the misdirection of fibers observed in the *Trkb^PLC/PLC ^*mutants. We show that calyx formation around type I hair cells in the vestibular sensory epithelia requires BDNF/TrkB signalling via the PLCγ docking site. In absence of this site, fibers show a limited ability to switch from growth to calyx formation.

Past research has convincingly shown that absence of any fibers at any time does not interfere with the maturation of recognizable type I and type II vestibular hair cells [20,22,34,35] implying that this process is cell autonomous. Indeed, we show here that the development of morphologic characteristics of type I hair cells does not depend on the formation of a calyx. Instead, we suggest that calyx formation is mediated through the PLCγ activated signalling pathway(s) downstream of the TrkB receptor. In the absence of this pathway(s), fibers cannot be converted in fully developed calyces and form bouton terminals instead. Of note is the fact that type I hair cells receive efferent terminals (Fig. 7) which are virtually excluded from these cells by the developed calyx in wild type mice, and suggest that formation of a calyx is directly related to the exclusion of direct efferent innervations on type I hair cells. It remains unclear what exactly are the differences in the BDNF release from type I and type II hair cells, both of which are strongly positive for BDNF in embryos [2,17,36]. Closer examination of the amounts of BDNF expressed in postnatal type I and type II hair cells is necessary to understand whether there is a differential expression of BDNF in one of the two differentiating hair cell types thereby contributing to the transformation of boutons into calyces via the TrkB/PLCγ signalling activated pathway(s). It is entirely possible that the downregulation of *Bdnf *expression in postnatal ears [37-39] is different in the two hair cell types and thus provides the proper signalling for afferent transformation into calyces.

In summary, these data support the notion that the TrkB/PLCγ site activated pathway(s) mediate structural synaptic plasticity in the PNS. Indeed, our data showing the lack of proper development of afferent calyces on differentiated type I vestibular hair cells demonstrate clearly that BDNF signalling through the TrkB/PLCγ site activated pathway(s) is an essential component of this structural synaptic plasticity.

## Methods

### Mouse strains

Generation of the TrkB point mutant mice has been described previously [5,13]. Briefly, the TrkB^PLC ^and TrkB^D ^mice were created using a cDNA "knock-in" approach [40]. Y816 (for the TrkB^PLC ^mice) or Y515 and Y816 (for the TrkB^D ^mice) were changed into phenylalanine in the mouse *Trkb *cDNA. Mutant *Trkb *cDNAs and wild-type *Trkb *cDNA (as a control for the strategy) were fused to the juxtamembrane exon of the mouse *Trkb *gene, and standard homologous recombination in ES cells (E14.1) was used to generate the targeted *TrkB^PLC^, Trkb^D ^*and *Trkb^WT ^*alleles.

### Histology and neuron counts

Mice were fixed by cardiac perfusion using 4% paraformaldehyde in 0.1 M phosphate buffer (pH 7.4). The heads were decalcified in 5% formic acid in phosphate-buffered saline for 4 days, embedded in paraffin, and serial sectioned at 8 μm in the horizontal plane. For cellular counts, the neurons within the vestibular ganglia were visualized using 0.02% cresyl violet. Neurons with a clear nucleus were counted in every 5 sections (40 μm intervals). The total number of neurons in one vestibular ganglion was then calculated by dividing the cell count by the number of sections counted, and multiplying this value by the number of sections containing the vestibular ganglion. The total number of neurons obtained for each of the two vestibular ganglia per animal were averaged and the result was corrected for split nuclei using Abercrombie's correction formula [41]. In the figure, the values represent the means and standard errors for each genotype. Mean neuronal counts were compared using unpaired t-tests or using one-way ANOVA followed by a Tukey's Multiple Comparison test (GraphPad Prism). The number of animals used is as follows: *Trkb^WT/WT^*, n = 3; *Trkb^PLC/+^*, n = 2; *Trkb^PLC/PLC^*, n = 5; *Trkb^D/D^*, n = 4; *Trkb^SHC/SHC^*, n = 2; *Trkb^-/-^*, n = 2.

### Circling behavior analysis

Mice were allowed to roam freely in their home cage whilst videos were recorded using a digital camera. A representative sample of the locomotor activity is shown.

**File Format: **MOV. Size: 16 MB. Playing the movie requires QuickTime.

### Immunohistochemistry

We used the rabbit KCNQ4 antibodies against KCNQ4 N- and C- termini followed by a goat anti-rabbit secondary antibody conjugated to Alexa Fluor 568 (Molecular Probes, Eugene, OR) as previously described [25]. Analyses using whole mount immunohistochemistry (IHC) and whole mount immunofluorescence (IF) were performed in the vestibular endorgans of mice at P0, P8 and P24. Due to low signal in the whole mount IF preparation of early postnatal vestibular tissue, we performed whole mount IHC and used the Vectastain^® ^ABC Kit (Vector Laboratories, Inc, Burlingame, CA). Images were taken with a Zeiss LSM 510 Meta NLO confocal microscope. The number of animals used is as follows: *Trkb^WT/WT ^*(P0 n = 3, P8 n = 3, P24 n = 2); *Trkb^PLC/PLC ^*(P0 n = 7, P8 n = 3, P24 n = 2); *Trkb^D/D ^*(P0 n = 2); *Trkb^SHC/SHC ^*(P0 n = 2).

### Lipophilic dye tracing

Mice at different stages were fixed by transcardiac perfusion with 4% PFA, and DiI lipophilic tracers (NV Maroon) were used to reveal afferent and efferent fibers of the inner ears as described [42]. Briefly, dyes were inserted into central target nuclei and left to diffuse to fill all profiles. Ears were subsequently dissected, mounted in glycerol and viewed with a Leica confocal microscope. The number of animals used for this experiment is as follows: *Trkb^WT/WT ^*(P0 n = 3, P8 n = 5, P24 n = 2); *Trkb^PLC/+ ^*(P8 n = 2, P24 n = 2); *Trkb^PLC/PLC ^*(P0 n = 8, P8 n = 6, P24 n = 2); *Trkb^D/D ^*(P0 n = 4); *Trkb^SHC/SHC ^*(P8 n = 2).

### Transmission Electron Microscopy and nerve diameter

Transmission Electron Microscopy (TEM) was preformed on osmicated ears, subdissected to allow serial sectioning of specific endorgans or nerves as previously described [9]. Thick sections were imaged in a compound microscope, while ultrathin sections were imaged using Hitachi or Jeol JEM 1230 TEM. Nerve numbers and diameters were counted and measured as previously described [9]; briefly, the diameter of the nerve to the posterior canal (PC) was measured using ImagePro software. The number of nerve fibers in the posterior vertical canal of P24, and P100 animals was determined by counting fibers on photographs taken at random throughout the nerve. The total number of fibers was then calculated using the measured area of the nerves. At least three sections at different levels of one canal were examined per animal. The number of animals used for this experiment is as follows: *Trkb^WT/WT ^*(P0 n = 2, P8 n = 2, P24 n = 6, P100 n = 6); *Trkb^PLC/+ ^*(P8 n = 1, P24 n = 4, P100 n = 6); *Trkb^PLC/PLC ^*(P0 n = 3, P8 n = 3, P24 n = 10, P100 n = 8).

### RNA isolation and Real-Time PCR

*Trpc *isoform expression in vestibular ganglion: total RNA was prepared according to [43]. RT-PCR reactions were performed according to [44] on total RNA from dissected vestibular ganglion of CF1 mouse inner ear. Positive controls consisted of cDNA templates derived from E15 mouse brain and retina. Negative controls consisted of no template or template without reverse transcriptase treatment. Gene-specific (gs) oligodeoxynucleotide primers were prepared for each of the *Trpc *genes (designed with the assistance of the Oligo 4.0 program, Natl. Biosciences, Plymouth, MN) as shown below (Table 1).

**Table 1:** Primer sequences for *Trpc* genes

Primer Designation	Primer Orientation	Primer sequence (5'-3')
mTrp1-2261	FOR	ATGACACCTTCCACTCGTTCATTGG
mTrp1-2871	REV	AGAAGTCCGAAAGCCAAGCAAATC
mTrp2com	FOR	AAAGACAGCACCGACCTCAGACC
mTrp2L-1025	REV	ACATTCCGCAGAGGACCACCTG
mTrp2L-2800	FOR	GATGTGGAATGGAAGTTTGCTCGC
mTrp2L-3349	REV	CCACAATCCCAGGCATAGTCAGC
mTrp2S-1654	REV	TCATTCACTCATCTTCCCAAACAGC
mTrp3-2819	FOR	AGAAGATAAGAGCCAAGCGACGG
mTrp3-3337	REV	GTCTCTCGTCTGCTCTTGGATGC
mTrp4-2463	FOR	CTCTACAATACAGTCAGCCAACGC
mTrp4-2463	REV	GTGTCTTCCACCACCACCTTCTC
mTrp5-3292	FOR	GGTGATGGACAGGAAGAACAAG
mTrp5-3922	REV	GAAGACAGGCAGGTTGATTAGC
mTrp6-2238	FOR	GGAATGTTCAACCTTTACTCCTAC
mTrp6-2921	REV	CTCATCGCTCTCCTTATCAATC
mTrp7-2616	FOR	TCAGCGAGAAGTTTGGGAAGAATC
mTrp7-3146	REV	GCTATCTGGGTTCTCATCTGGCAC
mTrp1-2261	FOR	ATGACACCTTCCACTCGTTCATTGG

Analysis of *Trpc3 *channel in vestibular organ of *Trkb *point mutants: mice of each genotype at 4 months of age (for *Trkb^+/+^*, n = 5 mice; for *Trkb^PLC/PLC^*, n = 6 mice and for *Ttrkb^WT/WT^*, n = 3 mice) were killed by cervical dislocation, then decapitated and their heads bisected. After removing skin and fat from the specimen, vestibular ganglions were dissected bilaterally. Briefly, brain material was slowly lifted up with forceps in order to visualize the inner ear and afferent fibers under a dissecting microscope (Leica Microsystems, USA). Total RNA from vestibular ganglions was extracted with TRizol reagent (Invitrogen, Paisley, UK), according to the instructions of the manufacturer. For first strand cDNA synthesis, 1 μg of total RNA was reverse-transcribed with the Ready To Go T-Primed first strand kit (Amersham Biosciences, NJ, USA), according to the manufacturer's instructions. All the samples were finally brought to a final volume of 200 μl in DEPC-treated water. PCR primers for the murine *Trpc3*, *Trpc4*, homologs and internal controls ubiquitin and NSE (Table 2) were designed based on published sequences in GenBank (for *Trpc3 *and *Trpc4 *primers see Table 1).

**Table 2:** Primer sequences for NSE and ubiquitin genes

Primer Designation	Primer Orientation	Primer sequence (5'-3')
m NSE	FOR	GTCCCTGGCCGTGTGTAAG
m NSE	REV	CATCCCGAAAGCTCTCAGC
m ubiquitin	FOR	TGGCTATTAATTATTCGGTCTGCAT
m ubiquitin	REV	CATCCCGAAAGCTCTCAGC

Real-Time PCR analysis was performed using the LightCycler 480 system (Roche, Portugal). The PCR reactions were performed using SYBR Green Jumpstart Taq ReadyMix (Sigma-Aldrich, UK) using 384 plates (Roche, Portugal) in a total volume of 20 μl: 10 μl of 2X SYBR Green I, 5 μl of primers mastermix (1.2 μm), 2,5 μl cDNA sample and water to 20 μl. Each sample was analyzed in duplicate. Thermal cycling was initiated with activation of the Jumpstart Taq DNA Polymerase by denaturation during 10 min at 95°C, followed by 45 cycles of a 15 s melting step at 95°C, a 15 s annealing step at 62°C, and a 10 s elongation step at 72°C (all temperature transition rates at 20°C/s). After amplification for 45 cycles, at least 10 cycles beyond the beginning of the linear phase of amplification, samples were subjected to a melting curve analysis according to manufacturer's instructions in order to confirm the absence of nonspecific amplification products and primer-dimers. Melting curves for, *Trpc3, Trpc4*, ubiquitin, and NSE showed one specific peak.

mRNA quantitative analysis: the mRNA levels of the constitutively expressed housekeeping gene encoding ubiquitin were used as a control in all experiments and final results were compared with the mRNA levels of NSE. The relative changes in the mRNA levels of TRPC in murine vestibular ganglions were determined using the ΔΔCp method as previously reported [45,46]. Accordingly, for each sample the "Crossing point" (Cp) values given by the LightCycler system II software, for each target gene, were subtracted by the respective Cp value determined for the ubiquitin gene for the same sample and condition (ΔCp). This allows normalizing changes in target gene expression. Afterwards, the ΔCp values were subtracted by the respective values of the control for the target gene giving ΔΔCp. The derivation to the formula 2-(ΔΔCp) sets each control at the unity since ΔΔCp (control) = 0.Statistical analysis was performed using one-way ANOVA followed by the Student's t test.

### Authorization for the use of experimental animals

EMBL-Monterotondo Animal Ethics Committee approved all animal procedures. We further confirm that all animal experiments conform to Italian and European regulatory standards.

## Abbreviations used in the document

AKT: v-akt murine thymoma viral oncogene homolog; BDNF: brain-derived neurotrophic factor; CamKII: calcium/calmodulin-dependent kinase II; DiI: 1,1'-dioctadecyl-3,3,3',3'-tetramethylindocarbocyanine perchlorate; FRS-2: FGF receptor substrate 2; IF: immunofluorescence; IHC: immunohistochemistry; KCNQ4: potassium voltage-gated channel subfamily KQT member 4; LTP: Long-term potentiation; MAPK: Ras/mitogen activated protein kinase; NSE: neuron-specfic enolase; PI3K phosphoinositide 3-kinase; PLCγ: phospholipase Cγ; PC: posterior canal crista; SHC: Src homology 2 domain-containing-transforming protein; TrkB/Ntk2: neurotrophin tyrosine kinase receptor B; *Trkb^-/-^*: mice carrying the germ-line null mutation for *Trkb*; TrkB^D^: mice with germ-line Y515F and Y816F point mutations in TrkB; TrkB^PLC^: mice with a germ-line Y816F point mutation in TrkB; TrkB^SHC^: mice with a germ-line Y515F point mutation in TrkB; TrkB^WT^: control mouse line for the TrkB^PLC ^mutant mice; TRPC3: Transient receptor potential cation channel, subfamily C, member 3.

## Competing interests

The authors declare that they have no competing interests.

## Authors' contributions

CS and BF planned and performed most of the experiments and, together with LM, drafted the manuscript. AB, KB and SR-S carried out the TrpC investigation and imaging of adult sensory epithelia. JH performed the behavioral analysis and imaging of the vestibular organs. LM conceived of the study, contributed to the experimental plans, provided theoretical input and supervision and wrote the manuscript. All authors read and approved the manuscript.

## Supplementary Material

Additional file 1 **Locomotor activity of a *Trkb^PLC/PLC ^mouse***. Movie demonstrating the circling behaviour of a *Trkb^PLC/PLC ^*mouse in its home cage. Note the higher basal locomotor activity compared to controls (*Trkb^WT/WT^*, additional file 2) and that circling occurs in both directions. Click here for file

Additional file 2 **Locomotor activity of a Trkb^WT/WT^mouse**. Movie demonstrating the locomotor behaviour of a *Trkb^WT/WT ^*control mouse in its home cage. Click here for file
